# Protocol for a Phase 2 randomized controlled patient-assessor blinded study: efficacy and safety of combined cortical and cerebellar dual-target transcranial magnetic stimulation for the treatment of essential tremor

**DOI:** 10.3389/fneur.2024.1505154

**Published:** 2025-01-08

**Authors:** Jing Xu, Na Cao, Yan Qu, Suhang Shang, Xincheng Liu, Xuexin Wang, Fangfang Hu, Xuerong Bai, Qiumin Qu, Meng Zhang, Hongmei Cao

**Affiliations:** ^1^Department of Neurology, The First Affiliated Hospital of Xi'an Jiaotong University, Xi’an, China; ^2^College of Mechanical Engineering, Xi'an Jiaotong University, Xi’an, China

**Keywords:** transcranial magnetic stimulation, essential tremor, dual-target stimulation, fMRI, neuromodulation

## Abstract

**Background:**

Essential tremor (ET) is the most common neurological movement disorder with few treatments and limited therapeutic efficacy, research into noninvasive and effective treatments is critical. Abnormal cerebello-thalamo-cortical (CTC) loop function are thought to be significant pathogenic causes of ET, with the cerebellum and cortex are common targets for ET treatment. In recent years, transcranial magnetic stimulation (TMS) has been recognized as a promising brain research technique owing to its noninvasive nature and safety. In this study, we will use left M1 cortex continuous theta-burst stimulation (cTBS) combined with right cerebellar hemisphere 1 Hz repetitive transcranial magnetic stimulation (rTMS) dual-target stimulation to explore the Safety, feasibility and efficiency of this dual-target stimulation mode, and the mechanism of its therapeutic effect.

**Methods:**

Twenty-four patients with ET will be randomly assigned to three groups: dual-target stimulation, single-target stimulation, or sham stimulation. The single-target stimulation group will receive stimulation of the right cerebellar hemisphere for 10 days, whereas the dual-target stimulation group will be given stimulation of both the left M1 cortex and the right cerebellar hemisphere. The sham stimulation group will be given sham stimulation for 10 days. Tremor will be assessed using both the subjective The Essential Tremor Rating Assessment Scale (TETRAS) and objective accelerometer-based tremor analysis. at baseline (before stimulation), after the first, fifth, tenth days of treatment (D1, 5, 10), 24 h after 10 days of treatment (D10-24 h), and 1, 2, 3, and 4 weeks after stimulation (W1, 2, 3, 4).

**Discussion:**

This is a Phase 2 randomized, controlled, patient-assessor blinded clinical trial. The goal of this study is to investigate the Safety, feasibility and efficiency of TMS for the treatment of ET.

## 1 Introduction

Essential tremor (ET) is one of the most common movement disorders affecting approximately 1% of the global population. The prevalence of ET increases by 74% every 10 years of age (1). ET is characterized by postural or action tremor in both upper limbs (2), and more than half of the patients report that tremor interfered with their everyday lives and jobs (3) and may become disabling (4). As China’s population ages, ET has become a chronic disease that can progressively worsen and affect the quality of life of patients (5–7).

The treatment for ET is limited (8, 9). Nothing available slows its progression, and the symptomatic drug benefit decreases with time. Propranolol and primidone are first-line medications with adverse effects, low patient compliance, and poor efficacy for refractory ET (10). With advancements in biomedical engineering and stereotactic technology, ET patients with severe symptoms can be treated with stereotactic ablation of the brain nuclei and deep brain stimulation (DBS) (11, 12). The ventral intermediate nucleus of the thalamus (Vim) (13, 14) is the most common target in treating ET, other potential targets, such as the subthalamic nucleus (STN) (15), the internal segment of the globus pallidus (GPi) (16), and the posterior subthalamic area (PSA) (17, 18) are also considered for their potential therapeutic benefits in DBS procedures. However, this treatment is invasive and costly (19), and the impact may fade with time (20).

Transcranial magnetic stimulation (TMS) has emerged as a noninvasive electrophysiological technique at the end of the twentieth century and has been used since its introduction (21). The TMS mechanism is as follows, a brief electric current is sent via a magnetic coil, generating a temporary high-intensity magnetic field that stimulates or inhibits parts of the brain beneath the coil (22). It is now widely utilized in the study, diagnosis, and treatment of neuropsychiatric diseases, such as neuropathic pain, depression, and Parkinson’s disease, but its use in ET treatment is still in the experimental stage (23).

Functional abnormalities of the cerebello-thalamo-cortical (CTC) loop are considered significant pathophysiological causes of ET (24), the cerebellum and cortex are currently common targets for ET treatment. TMS is a noninvasive and safe neuromodulation modality, has been used in several studies in the past. The use of repetitive transcranial magnetic stimulation (rTMS) (25–29) and continuous theta burst stimulation (cTBS) (30–33) to treat ET has been studied in several studies; However, given the different study designs, number of included cases, and stimulation modes, there are no consensus results. In this randomized, controlled, patient-assessor blinded clinical trial, we will use the left primary motor cortex (M1) area combined with the right cerebellar hemisphere as our dual-target transcranial magnetic stimulation, to preliminarily observe the safety, feasibility and efficiency of this stimulus pattern and to discover the possible mechanism of TMS on ET, with the aim of informing future larger-scale studies. We hope that dual-target stimulation will improve the stability of study treatment results.

Accordingly, we hypothesize that dual-target TMS therapy for ET is not only safe and feasible but also effective in alleviating tremor symptoms by modulating specific neural pathways.

## 2 Methods

### 2.1 Study design

This is a Phase 2 clinical trial designed to evaluate the efficacy and safety of dual-target TMS for the treatment of ET. The study is randomized and controlled, incorporating blinding for both patients and assessors. Patients will be screened for study participation within study enrollment. All participants will be evaluated via neurological examination, The Essential Tremor Rating Assessment Scale (TETRAS), electromyographic and accelerometric recording, and magnetic resonance imaging (MRI). The participants will be then randomly assigned to one of three groups: dual-target stimulation group, single-target stimulation group or sham stimulation group. We will examine the patients’ vital signs before and after each day of treatment and record daily treatment completion and adverse event occurrence. Electrophysiological detection and TETRAS will be performed at baseline, after the first, fifth, tenth day of treatment (D1, 5, 10), 24 h after 10 days of treatment (D10-24 h), and at 1, 2, 3 and 4 weeks (W1, 2, 3, 4) after stimulation.

MRI will be carried out at baseline, D10, and W4 (Table 1). Recruitment for this study is ongoing at the time of submission.

**Table 1:** Study schedule.

Procedure	Screening	Treatment				End	Follow-up			
Time point	D 0	D1-B	D1-A	D5-A	D10-A	D10-24 h	W1	W2	W3	W4
Basic										
Demography	✓									
Eligibility	✓									
Vital signs	✓	↔				✓	✓	✓	✓	✓
Informed consent	✓	↔				✓	✓	✓	✓	✓
Assessment										
TRETRAS		✓	✓	✓	✓	✓	✓	✓	✓	✓
Tremor analysis		✓	✓	✓	✓	✓	✓	✓	✓	✓
MRI	✓				✓					✓
Trial evaluation										
Adverse event			↔			✓	✓	✓	✓	✓
Safety evaluation			↔			✓	✓	✓	✓	✓

TRETRAS, The Essential Tremor Rating Assessment Scale; MRI, Magnetic Resonance Imaging; D0, Days before treatment; D1-B, Before the first day of treatment; D1-A, After the first day of treatment; D5-A, After 5 days of treatment; D10-A, after 10 days of treatment; D10-24 h, 24 h after 10 days of treatment; W1, One week after treatment completion; W2, Two weeks after treatment completion; W3, Three weeks after treatment completion; W4, Four weeks after treatment completion.

### 2.2 Participants and recruitment

Participants will be recruited from the Department of Neurology, the First Affiliated Hospital of Xi’an Jiaotong University, with reference to the diagnostic criteria for ET established by the International Movement Disorders Society in 2020 (6); The diagnosis of ET will be made independently by two professors specializing in movement disorders in the Department of Neurology. All the participants should sign informed consent. The study protocol is approved by the Ethics Committee of the First Affiliated Hospital of the Xi’an Jiaotong University.

#### 2.2.1 Inclusion criteria

1. Males or females, ages from 18 to 80 years;
2. Meet the diagnostic criteria for ET with visible upper limbs tremor;
3. The participants are qualified to complete the scales and perform a physical neurological examination. They should not receive any anti-tremor therapy during the stimulation period;
4. Signed written informed consent.

#### 2.2.2 Exclusion criteria

1. Participants with severe neuropsychiatric disease such as schizophrenia, bipolar disorder, and major depressive disorder.
2. Have a clinically significant abnormality on the screening examination that might affect safety, study participation, or confound the interpretation of study results;
3. Participants with contraindications to MRI, such as those with metal implants in their bodies;
4. Severe migraine, cranial injury, suicide risk, or epilepsy risk.

### 2.3 TMS stimulation

#### 2.3.1 Dual-target stimulation

The left M1 and right cerebellar hemisphere will be selected for dual-target stimulation. The resting motor threshold (RMT) and active motor threshold (AMT) will be assessed during the first treatment session. The patient will be positioned supine, with fingers relaxed and palms facing upward. Preliminary stimulation will be administered at 70% of the maximum output intensity of TMS, while observing the contraction of the contralateral thumb (abductor pollicis brevis) to identify the optimal stimulation site capable of eliciting motor-evoked potentials (MEP) in the abductor pollicis brevis muscle. The stimulation intensity will then be gradually decreased to determine the RMT, defined as the minimum stimulation intensity required to produce a slight contraction of the contralateral abductor pollicis brevis in 5 out of 10 attempts (with evoked thumb abductor potentials of 50 microvolts amplitude or more) (33–35). While AMT is defined as the lowest stimulus intensity that elicited a peak MEP amplitude of ≥200 microvolts amplitude during a 10% Maximum Voluntary Contraction (MVC) of the dorsal interosseous muscle of the first interosseous (right-hand side). This criterion must be met in at least 5 consecutive trials out of 10 (36, 37).

A figure-eight coil will be utilized in conjunction with a magnetic-field stimulator (Mag TD 40, Factory No. 44777533). The specific operational procedures will be conducted in accordance with the TMS operation guide (38). The operator will consist of qualified TMS treatment professionals from the First Affiliated Hospital of Xi’an Jiaotong University, who have undergone both theoretical and practical training, enabling them to independently administer TMS treatment to a designated number of patients.

In this study, we will localize the left M1 area according to the international Electroencephalogram (EEG) 10–20 system electrode placement, the specific positioning is shown in Table 2. cTBS uses triplets of stimuli at 40 Hz, repeated every 200 ms for 40 s. The stimulation parameters for the right cerebellar hemisphere are as follows, over a period of 10 days, a total of 90 repetitions of 900 pulses will be administered at a frequency of 1 Hz, with each stimulation period lasting 10 s followed by a 2-s interval. We will apply 1 Hz rTMS to stimulate the right cerebellum, followed by cTBS to stimulate the M1 region in a sequential intervention. To ensure accurate stimulation, the patient will be positioned supine on the treatment bed, maintaining a still head position while the device is securely fixed on the head above the designated stimulation target.

**Table 2:** Location of the stimulus target.

Stimulus target	Location
Left M1 region	Above the left cerebral hemisphere M1 area (C3, International electroencephalogram 10–20 system), perpendicular to the central sulcus (45° from the midline of the hemisphere)
Right cerebellar hemisphere	1 cm below the occipital eminence, 3 cm to the right parietal

In rTMS studies, stimulation intensity is based on the Motor Threshold (MT), typically ranging from 80 to 120% of RMT to achieve desired neural effects safely (39). For cTBS, the standard intensity is 80% of the AMT, effectively inducing long-term inhibitory effects while ensuring participant safety (40). We select a stimulation intensity of 90% of the RMT for 1 Hz rTMS and 80% of the AMT for cTBS, based on previous research (26, 27, 29, 32, 33).

#### 2.3.2 Single-target stimulation

Patients assigned to the single-target stimulation group will receive real stimulation to the right cerebellar hemisphere and sham stimulation to the left M1 cortex, utilizing the same methods and stimulation parameters as previously described.

#### 2.3.3 Sham stimulation

Patients assigned to the sham-stimulation group will receive sham stimulation targeting the left M1 cortex and the right cerebellar hemisphere. This procedure mimics the characteristics of actual stimulation by reversing the coil by 90 degrees; however, it does not produce an effective stimulus.

To ensure the rights of our participants, we will provide an additional 10 days of open-label right cerebellar hemisphere rTMS to all groups 1 month after the completion of all interventions and the four-week follow-up period, as a form of ethical compensation. Informed consent will be obtained from all participants prior to the initiation of the study.

### 2.4 Accelerometer-based tremor analysis

Patients will be positioned in a comfortable chair equipped with armrests, and both upper limb tremor movements will be recorded utilizing a multi-sensor signal accelerometer (chip model: MPU6500). The device will be affixed to the dorsal surface of the metacarpal bones of both hands, enabling the capture of resting tremor (the patient will be instructed to relax and rest their hands on the chair armrests), postural tremor (two specific poses will be required: one with the arms extended forward and parallel to the ground, and the other maintaining a wing-beat posture), and kinetic tremor (assessed through the finger-nose test). Each movement will be recorded for 20 s, with tremor measurements repeated twice at each time point. The average of the two recordings will subsequently be calculated.

### 2.5 Brain imaging

Resting-state functional magnetic resonance imaging (Rs-fMRI) images, three-dimensional (3D) T1-weighted images, and conventional T2-weighted images will be acquired using a 3.0 Tesla MRI system (Philips, Netherlands, Model: Ingenia 3.0T CX, Serial Number: 78356). Foam pads provided by the manufacturer will be employed to minimize motion-related interference, and earplugs will be inserted into both external auditory canals to mitigate auditory stimulation. During the scanning procedure, participants will be instructed to keep their eyes closed and remain awake.

Rs-fMRI will be conducted utilizing an echo-planar imaging (EPI) pulse sequence with the following parameters: 48 slices, slice thickness/gap of 3.5/0 mm, repetition time (TR) of 2,000 ms, echo time (TE) of 30 ms, flip angle (FA) of 90°, and a field of view (FOV) of 256 × 256 mm. Additionally, 3D T1-weighted images will be acquired with parameters including the shortest TR and TE, a flip angle of 12°, a FOV of 256 × 256 mm, a matrix of 256 × 256, and a total of 194 slices. Conventional T2-weighted images will also be obtained, characterized by a TR of 3,000 ms, a TE of 80 ms, a slice thickness/gap of 5.0/1 mm, a FOV of 230 × 209 mm, and a total of 27 slices. It is important to note that while the conventional T2-weighted images will not be utilized for data processing, they will be employed for image evaluation purposes.

All functional imaging data preprocessing will be conducted utilizing the Statistical Parametric Mapping (SPM12) and Data Processing & Analysis for Brain Imaging (DPABI) software, both of which operate on the Matrix Laboratory platform (Matlab Release 2023b). The main steps of image preprocessing are as follows: (i) the initial 10 volumes will be removed to mitigate T1 equilibrium effects; (ii) slice-timing correction will be applied; (iii) subsequent functional images will be realigned to the first volume to correct for within-run head motion; (iv) T1-weighted images will be co-registered to the mean Rs-fMRI data for each subject. Specifically, SPM Diffeomorphic Anatomical Registration Through Exponentiated Lie Algebra (DARTEL) segmentation will be employed to partition the 3D T1-weighted images into probability maps for gray matter (GM), white matter (WM), and cerebrospinal fluid (CSF). All GM, WM, and CSF images will be resampled and normalized to the Montreal Neurological Institute (MNI) space. (v) Nuisance covariates, including WM signal, CFS, and the mean time series of the entire brain, will be regressed out; (vi) first-order polynomial functions will be utilized to eliminate linear trends; (vii) a filter will be applied to reduce low-frequency drift and high-frequency noise; and (viii) smoothing will be performed (41, 42). Functional connectivity (FC) analysis will be based on the pre-processed images.

This study will employ Independent Component Analysis (ICA) to assess the FC of CTC circuits, the Default Mode Network (DMN), and additional networks, including executive, frontoparietal, auditory/language, and visual networks. FC can be evaluated by analyzing the correlation of time-varying signals from two spatially distinct regions. Correlation analyses will also be performed (29, 43, 44). The objective is to compare alterations in brain functional networks across different groups and to examine changes in the strength of FC before and after treatment, thereby investigating the modulatory effects of TMS on brain networks.

### 2.6 Termination

Intervention may be concluded at an appropriate juncture based on the following circumstances: (i) patients who experience serious adverse events (SAEs) during treatment, including but not limited to tinnitus, dizziness, headache, localized discomfort at the site of irritation, or seizures; (ii) patients who request termination for reasons unrelated to the treatment; (iii) patients who identify a significant disease that was not previously detected before the initiation of treatment, or who develop other emerging health issues during the treatment process; (iv) upon the completion of all scheduled treatments.

Throughout the treatment process, we will implement health education for patients and their families, while accommodating treatment schedules to minimize disruptions to their daily lives. Additionally, we will prioritize patient feedback to enhance their trust, thereby improving adherence to the treatment regimen.

### 2.7 Outcome measurement

#### 2.7.1 Primary outcome

The primary outcome measure will be the mean change from baseline over the course of 4 weeks (at days 1, 5, 10, D10-24 h, then at weeks 1, 2, 3, and 4). This assessment will focus on the average tremor score derived from the TETRAS. The TETRAS (45) is a widely utilized instrument for evaluating the severity of ET and is employed to assess the remission and recovery of ET following TMS. The scale comprises a 12-item section that evaluates activities of daily living and a 9-item section that assesses tremor severity. Each item is rated on a scale from 0 to 4, with higher scores indicating greater severity.

#### 2.7.2 Secondary outcomes

The secondary outcomes encompassed the mean changes in tremor power, amplitude, and frequency as recorded by accelerometers, in addition to the adherence rate and the incidence rate of SAEs.

During the 10-day stimulation period, a delay of up to 2 days will be permissible. Participants who experience a delay exceeding 2 days but less than 10 days will be classified as noncompliant. The compliance rate is defined as the percentage of participants who complete the 10-day stimulation within a maximum of 12 days. A high adherence rate suggests that the patient has completed the entire course of treatment punctually and indicating greater feasibility of the intervention.

Adverse event (AE) is defined as any adverse medical occurrence that occurs in an individual during clinical research, irrespective of whether it is attributable to medication or intervention. SAEs can lead to fatal outcomes for patients. A lower rate of SAEs indicates a higher level of safety associated with the intervention. A SAE is specifically defined as an event that results in one of the following outcomes for a participant: death, life-threatening conditions, hospitalization, disability, or permanent damage or necessitates intervention to avert permanent impairment or damage (in the case of devices), as well as other significant medical events.

#### 2.7.3 Exploratory outcomes

Alterations in brain FC (as measured by total integration) across various groups. Additionally, changes in the strength of FC before and after treatment will serve as exploratory outcome measures to explore the mechanisms underlying TMS treatment for ET. MRI will be conducted at baseline, on D10, and at W4.

### 2.8 Sample size

This pilot study involves a small number of patients and aims to assess the practicality and feasibility of the methodologies intended for use in a subsequent, larger, and more comprehensive investigation, as well as to identify any unforeseen challenges. Badran (46) indicated that a sample size of five patients per group is sufficient to ascertain whether a larger multicenter trial should be pursued. Additionally, Popa (29) proposed that 11 participants per group are adequate for evaluating feasibility in a pilot study. Drawing on prior research (28), our objective is to recruit eight patients per group. The findings of this study are expected to provide preliminary insights into the safety and feasibility of dual-target TMS in patients with ET and will inform the estimation of sample size and the conduct of a power calculation for the planning of a Phase 3 trial.

### 2.9 Randomization

In this study, a simple randomization method will be used to generate 30 random numbers utilizing a table of random numbers, commencing from row 2 and column 3, with every two adjacent numbers considered as a single random number. These random numbers will be divided by three to determine the remainder, which will categorize participants into three groups: a remainder of zero for the double-target group, one for the single-target group, and two for the sham stimulus group. The pre-generated random numbers will be securely placed in opaque, sealed envelopes, which will be stored and maintained by the lead investigator. At the initiation of treatment, one envelope will be opened to ascertain the group assignment of participants in the order of their enrollment. Throughout the duration of the study, both the participants and the assessors will remain unaware of the group assignments.

### 2.10 Blinding

This study will follow a patient-assessor blinded clinical design. To enhance the validity of the stimulation procedures, all participants will undergo two rounds of stimulation, each targeting both the left M1 region and the right cerebellar hemisphere. In the dual-target stimulation group, during the first round, actual stimulation will be administered to the cerebellum while the cortical coil will be oriented at a 90° angle to prevent effective stimulation; during the second round, this approach will be reversed. The single-target stimulation group will also go through two rounds, with the first round focused on true cerebellar stimulation and the other coils flipped. To maintain patient blinding, the sham stimulation group will alter the orientation of all coils during both rounds.

Blinding may be deemed appropriate under the following circumstances: (i) the successful completion of the entire trial; or (ii) the occurrence of serious adverse reactions during the study. In such instances, the treatment personnel are required to promptly report the adverse reactions to the principal investigator, who will determine whether to disclose the blinding.

## 3 Date management and monitoring

Data will be collected by treatment operators and outcome raters, with participants receiving training prior to the commencement of the trial to ensure the quality of the data. The endpoint assessor will document and authenticate the data on a case report form (CRF), which will remain unaltered; any modifications to the CRF will be signed and dated. The scale assessor will evaluate the data in accordance with standardized norms and recorded videotapes. Following the collection and entry of data into the electronic data collection system, an additional researcher will review the data to verify its accuracy. Once the validity of the data input is confirmed, the database will be locked; the individual responsible for the subject will have the ability to view and modify the data, while access will be restricted for others.

The Data Monitoring Management Committee, which is composed of members from the Department of Scientific Research at the First Affiliated Hospital of Xi’an Jiaotong University, is responsible for overseeing the data. This committee operates as an independent third party, separate from both the investigator and the sponsor, and maintains no conflicts of interest in relation to this study. The committee will conduct a comprehensive analysis of the research data biannually to ensure its quality.

## 4 Safety monitoring

Prior to the experiment, investigators will inform all enrolled patients of the possible benefits and risks and have them sign an informed consent form.

TMS may lead to adverse events, including headache, localized discomfort at the stimulation site, hearing loss, tinnitus, seizures, and syncope. Consequently, several preventive measures are recommended: the use of earplugs during TMS therapy, discontinuation of treatment in the event of headache or localized discomfort, careful monitoring of symptom changes, and the administration of analgesics as needed. Although epileptic seizures are rare during TMS, if seizures or syncope occur, it is imperative to immediately cease stimulation, assess the patient’s vital signs, and conduct further medical evaluations after the seizure has resolved. In instances of discomfort during the procedure, treatment should be halted immediately, followed by clinical observation and symptomatic management (47).

The Ethics Committee of the First Affiliated Hospital of Xi’an Jiaotong University will oversee the safety of this study and provide guidance as needed. In the event of an adverse occurrence, subjects must receive prompt medical attention. Detailed documentation of the adverse event—including its name, time of occurrence, duration, severity, therapeutic measures undertaken, and treatment outcomes—should be meticulously recorded and reported to the Research Safety Supervisory Committee in a timely manner. If necessary, blinding procedures will be implemented in accordance with established protocols. The attending physician will evaluate the causal relationship between the treatment interventions and any adverse outcomes, and will determine whether the patient should continue with follow-up treatment based on the specific circumstances.

## 5 Statistical analysis

In this study, we will conduct an analysis of the data utilizing the principles of modified intention-to-treat (ITT) analysis, which will encompass patients who have completed a baseline assessment of the primary outcome indicators. Safety analyses will be performed on all patients who have received at least one session of TMS. Continuous variables will be expressed as the mean ± standard deviation (SD) or median (25th percentile, 75th percentile), while categorical variables will be presented as percentages (%). For the assessment of baseline characteristics, continuous variables will be analyzed using One-way ANOVA (normal distribution) and Kruskal-Wallis H test (non-Gaussian distributions). Categorical variables will be evaluated using Chi-square or Fisher’s exact tests. The primary outcome measure will be the mean change from baseline over a period of 4 weeks, assessed at days 1, 5, 10, D10-24 h, then at weeks 1, 2, 3, and 4, specifically focusing on the average tremor score derived from the TETRAS. We will employ generalized estimating equations (GEE) for repeated measures within the modified ITT population. Additionally, sensitivity analyses will be conducted on the primary outcome, considering age, sex, disease duration, and baseline tremor score as covariates. Effects will be expressed as estimates along with 95% confidence intervals (CIs). GEE will also be applied to analyze secondary outcome measures, including the mean change in tremor power, amplitude, and frequency as recorded by accelerometers.

For data that is randomly missing between TMS sessions, where the missing data constitutes less than 5% of the sample, we will employ the last observation carried forward method to impute the missing values. Additionally, sensitivity analyses will be conducted to evaluate the impact of these imputations on the study outcomes. Conversely, missing data resulting from early dropouts (before 4 weeks) will not be imputed, as this data is not considered to be missing at random. Instead, these instances of missing data will be censored at the date of the participant’s last visit. All statistical analyses will be performed using IBM SPSS version 27.0, and *p*-values less than 0.05 will be considered statistically significant (two-tailed).

## 6 Discussion

The objective of this study is to examine the safety, efficacy, and feasibility of dual-target TMS therapy for the treatment of ET. While this investigation will primarily function as a pilot study for a larger multicenter trial, it aims to explore novel therapeutic possibilities for ET.

Dual-target TMS shows great potential in the treatment of neurological diseases. A study exploring the efficacy of combined TMS targeting the cerebellum and the M1 in patients with tremor-dominant subtypes of Parkinson’s disease (T-PD) found that dual-target rTMS significantly outperformed single-target rTMS in alleviating tremor symptoms, enhancing quality of life. While this study focuses on T-PD, it also raises the potential application of dual-target rTMS to treat tremor symptoms in other contexts (48). Furthermore, dual-target TMS has been shown to be more effective than single-target stimulation in facilitating symptomatic improvement in conditions such as chronic tinnitus and post-stroke cognitive impairment (49, 50). Abnormalities in the CTC loop play a significant role in the pathogenesis of ET, dual-target TMS can simultaneously affect multiple nodes in the CTC circuit involved in ET, potentially achieving a more comprehensive therapeutic effect.

cTBS uses rapid pulse patterns to quickly inhibit targeted brain areas (51), while low-frequency rTMS provides more lasting effects, suitable for long-term modulation (52). We hypothesize that combining these TMS techniques can yield complementary benefits. Chuang et al. (32) found that cTBS suppresses motor cortex excitability in ET patients and reduces tremor amplitude. Batra et al. (33) showed that cTBS significantly improves tremor symptoms by increasing the contralateral silent period (cSP). Gironell et al. (25) and Popa et al. (29) reported tremor improvements after rTMS stimulation of the cerebellum. However, data on the effects of noninvasive brain stimulation techniques applied to the cerebellum and motor areas of ET patients show variability in their effects on tremor (53, 54). Specifically, there is a significant degree of variability among individuals. Olfati et al. (28) found no significant improvement in the overall tremor score with rTMS compared to sham stimulation on days 5, 12, or 30 after treatment. In contrast, Popa et al. (29), using the same stimulation protocol, observed a significant and lasting reduction in all aspects of tremor. It is important to consider that ET is a highly heterogeneous disorder with potential inter- and intra-individual differences, where factors such as anxiety (55), fatigue (56), and caffeine consumption (57) can all influence the severity of tremors. Furthermore, the placebo effect is a concern that should not be overlooked; Shin et al. (27) found that there was an improvement in tremor scale scores regardless of whether the stimuli were real or sham. The cerebellum and cortex are the most popular targets for stimulation in TMS studies aimed at treating ET. Previous findings suggest that the outcomes of TMS for ET are highly variable, and we speculate that the use of double-target stimulation may enhance the stability of treatment effects.

In this study, we will use Rs-fMRI to explore the alterations in brain functional connectivity before and after TMS treatment for ET, as well as to assess whether FC differs among three distinct groups. Studies have shown disruption in FC between the bilateral cerebellum and the cortex in patients with ET (58, 59). Furthermore, tremor severity may exhibit a positive correlation with FC between cerebellar lobule VI and VIM, as well as between the VIM and the cortex (60). These findings underscore the critical role of the CTC loop in the pathogenesis of ET.

Gironell et al. found a significant reduction in tremor scores 5 min after rTMS, but the effect disappeared 60 min after stimulation. Low-frequency rTMS may temporarily affect tremor production by modulating cerebellar output (25). Popa et al. found that cerebellar rTMS restored FC in the CTC network after five consecutive days of treatment (29). Previous studies have shown that patients with ET have increased tremor-related activity in several regions of the cerebellum (61). TMS applied to the cerebellum modulates synaptic plasticity and thus restores normal function of circuits involved in motor control in the cerebellum (62). While cTBS may reduce the excitability of the M1 cortex by inducing long duration depression (LTD), thereby decreasing tremor amplitude (33). Several studies have shown that ET is associated with abnormal function of the inhibitory neurotransmitter γ-Aminobutyric Acid (GABA) (32, 63, 64), GABA levels in the CSF have been observed to be decreased (65). Patients with ET exhibit heightened 11C-Flumazenil binding to GABA receptors within the ventrolateral thalamus, dentate nucleus, and pre-motor cortex (66). In our study, we will further explore the mechanism of TMS for ET using MRI.

It was found that cTBS applied to either the motor cortex or the cerebellum resulted in a reduction of MEP in healthy control subjects, but not in individuals with ET (30, 31). A potential explanation for this discrepancy is that the cerebellar-brain inhibition (CBI) may be altered in patients with ET. Following the administration of TMS to the cerebellum, if TMS is subsequently applied to the motor cortex within a 5–7 ms interval, a reduction in motor cortex-evoked MEPs is observed; this phenomenon is referred to as CBI (67), which is believed to be mediated by enhanced inhibition of Purkinje cell output to the dentate nucleus and thalamus (68). In addition, the cerebellum plays a central role in the pathogenesis of ET (69), we intend to first stimulate the cerebellum and subsequently the cortex to further investigate this mechanism from an imaging perspective.

In summary, TMS may serve as a valid tool for the treatment of ET and for modulating the CTC pathway, we use a combination of cortical and cerebellar stimulation, as suggested by evidence-based guidelines. Conducting a study on dual-target stimulation holds promise for improving patient outcomes. Furthermore, MRI has been utilized to investigate the pathogenic pathways associated with ET, providing insights into the underlying mechanisms of this condition. Such advancements have the potential to substantially enhance the quality of life for individuals affected by ET, enabling them to regain independence and participate in daily activities.
